# Circulating serum sVCAM-1 concentration in advanced ovarian cancer patients: correlation with concentration in ascites

**DOI:** 10.2478/raon-2013-0066

**Published:** 2014-07-10

**Authors:** Marina Jakimovska, Katarina Cerne, Ivan Verdenik, Borut Kobal

**Affiliations:** 1 Department of Obstetrics and Gynaecology University Medical Centre, Ljubljana, Slovenia; 2 Department of Pharmacology and Experimental Toxicology, Faculty of Medicine, University of Ljubljana, Ljubljana, Slovenia

**Keywords:** sVCAM-1, ovarian cancer, flow cytometry

## Abstract

**Background:**

Vascular cell adhesion molecule-1 (VCAM-1) is associated with ovarian cancer progression but the origin of its soluble form (sVCAM-1) in serum is not well investigated. The purpose of this study was to elucidate whether the concentration of sVCAM-1 in serum correlates with the concentration in ascites, that represents local tumour environment, and with systemic inflammation, various clinicopathological characteristics, and patient outcome.

**Patients and methods.:**

Thirty-six patients with advanced ovarian cancer were included in the study. Serum for sVCAM-1 analysis was obtained prior to surgery. Ascites samples were collected at the beginning of the operation. Clinical data were collected from patients’ medical records. sVCAM-1 in samples was analysed by flow cytometric bead-based assay. The mean follow-up period was 11 months (range 0–23) from the time of surgery.

**Results:**

Serum sVCAM-1 concentrations are positively correlated to ascites sVCAM-1 concentrations. There was a weakly positive correlation of serum sVCAM-1 with tumour size and no correlation with inflammatory tumour markers, FIGO stage or grade. Higher concentrations of sVCAM-1 were associated with poor disease outcome (death from ovarian cancer) in almost all cases before chemotherapy was started.

**Conclusions:**

This is the first study demonstrating that serum concentrations of sVCAM-1 in advanced ovarian cancer patients correlate with sVCAM-1 concentrations in ascites, thus expressing the biologic potential of malignant disease to metastasis, rather than systemic inflammation. Higher serum and ascites sVCAM-1 concentrations might have predictive potential for different biologic behaviour.

## Introduction

Ovarian cancer (OC) is relatively asymptomatic in the early stages; over 70% of all OC cases are diagnosed at advanced stages, with extensive seeding of the peritoneal cavity by tumour cells associated with ascites.^1^,^2^ Many promising bio-markers have been studied in serum and plasma of OC patients in order to provide early diagnosis and disease monitoring, but only carbohydrate antigen 125 (CA125) is approved in clinical use, and human epididymis protein 4 (HE4) that has been approved only as aid for monitoring patients.^3^–^8^ CA125 and HE4 are far from ideal, since both have limitations in their specificity and sensitivity.^7^ An excellent body fluid for discovering potentially more specific biomarkers is the local tumour environment represented by ascites, due to the presence of tumour cells and soluble proteins, reflecting the secretome of the tumour environment.^4^,^9^–^11^ It has been proposed that ascites is crucial for OC progression by favouring the dissemination of OC cells, which are detached from the primary tumour, within the peritoneal cavity. Transcoelomic dissemination is the most common manner of OC spread.^12^,^13^

A large proportion of soluble proteins in the acellular fraction of ascites have been found to be related to the processes of adhesion and cell movement.^14^ The disseminated OC cells preferentially adhere to the mesothelium, a layer of mesothelial cells that lines the peritoneum and surrounds the serosal surface of organs in the abdominal cavity. Vascular cell adhesion molecule-1 (VCAM-1), expressed in activated mesothelial cells, has been identified as an important mediator for adhesion of OC cells to and invasion through the mesothelium.^15^ Although the membrane-bound form is difficult to measure *in vivo*, the soluble form of VCAM-1 (sVCAM-1) can be detected in ascites. However, its concentration in ascites of OC patients has not previously been measured.

sVCAM-1 was analysed in the serum of OC patients, and an association with tumour presence was reported.^16^–^18^ Interpretation of detected levels of sVCAM-1 in serum in terms of its importance as a biomarker is hindered by lack of knowledge as to what factors determine the steady-state levels of this adhesion molecule in serum. Since VCAM-1 is involved in inflammatory reaction and systemic inflammatory response in cancer patients is a common phenomenon, there is a need to elucidate whether detected concentrations of sVCAM-1 in serum are truly cancer-specific.^19^

The aim of this study was to elucidate whether concentrations of serum sVCAM-1 detected in OC patients correlate with sVCAM-1 concentrations in the local tumour environment represented by ascites, thus expressing the biologic processes of malignant disease. To evaluate whether serum concentrations of sVCAM-1 are related to systemic inflammation, we investigated the relationship between concentrations of sVCAM-1 and markers of inflammation (C-reactive protein (CRP) and total white blood cells (WBC)). Additionally, we examined the relationship of sVCAM-1 concentrations to various clinicopathologic variables, as well as to patient outcome.

## Patients and methods

### Patients

Thirty-six patients with stage III and IV primary ovarian cancer, operated in two years period of time (from 2011 to 2012) at the Department of Gynecology, University Medical Centre Ljubljana, were included in the study. Staging of the disease was according to the International Federation of Gynecology and Obstetrics (FIGO) classification for staging ovarian cancer. CRP value, WBC count, data on tumour histological type, grade and primary tumour size were collected from patients documentation. Prior to analysis, all patients received detailed oral and written information about the research and procedures, and they signed informed consent for analysis of their blood and ascites for the purposes of the research. The trial was approved by the National Medical Ethics Committee of Republic of Slovenia with number of approval 82/01/11 and was in agreement with the Helsinki Declaration.

### Collection and storage of samples

Venous blood samples were obtained prior to surgery while the patients were hospitalized for pre-operative preparation. Four ml of peripheral blood were collected into a vacutainer, without anticoagulant or other additives. Serum was separated by centrifugation at 2000 x *g* for 15 minutes at 4°C. Blood for full blood count and CRP was obtained at the same time as for sVCAM-1 analysis. At the beginning of the operation, immediately after entry to the abdominal cavity, twenty ml of ascites were aspirated into a sterile syringe and immediately transferred into a conical tube, which was kept on ice until centrifugation at 1000 x *g* for 10 min at 4°C within 30 minutes. Sera and supernatant of ascites were stored in aliquots at −80°C. No more than 2 freeze-thaw cycles were allowed for any sample.

### Analysis of sVCAM-1 with flow cytometric bead-based assay

Concentrations of sVCAM-1 in samples were measured using a FlowCytomix Simplex Kit (eBio-science, Vienna). The kit consists of fluorescent microspheres (diameter: 4 μm, emission wavelength at 700 nm) coated with specific antibodies raised against sVCAM-1. It also contains a biotin-conjugated second antibody and straptavidine-phycoerythrin emitting at 575 nm. Samples were run on a Cell Lab Quanta^TM^ SC-MPL (Beckman Coulter). Samples were acquired by the Cell Lab Quanta^TM^ SC-MPL software (Beckman Coulter) and analysed using Flowcytomix^TM^ Pro 3.0 software (eBioscience). Electronic volume *vs*. side scatter gating was employed to exclude any sample particles other than 4 μm microspheres. A seven point standard curve ranging from 2.74 to 2000 ng/ml was obtained by serial dilution of the reconstituted lyophilized standard. The lower limit of detection was 0.9 ng/ml.

### Statistical analysis

Data are presented as mean ± SD. The normality of distribution was tested with the Kolmogorov-Smirnov test. Pearson’s correlation coefficient was used to calculate the strength of the relationship between normally distributed variables. Since data for tumour size was non-normally distributed, Spearman’s correlation coefficient was used to calculate the strength of the relationship between serum sVCAM-1 and tumour size. An independent samples t-test, and Pearson’s chi-square test were used to compare variables between patients that died before the start or during the adjuvant chemotherapy and those who finished complete treatment and are still living. A *p* of <0.05 was considered significant. Statistical analysis was performed using software statistical package SPSS, version 19 (IBM Statistics, USA).

## Results

The clinical characteristics of the investigated patients are summarized in Table 1.

The data were approximately normally distributed for all variables included in the analysis, except for tumour size.

**FIGURE 1.**
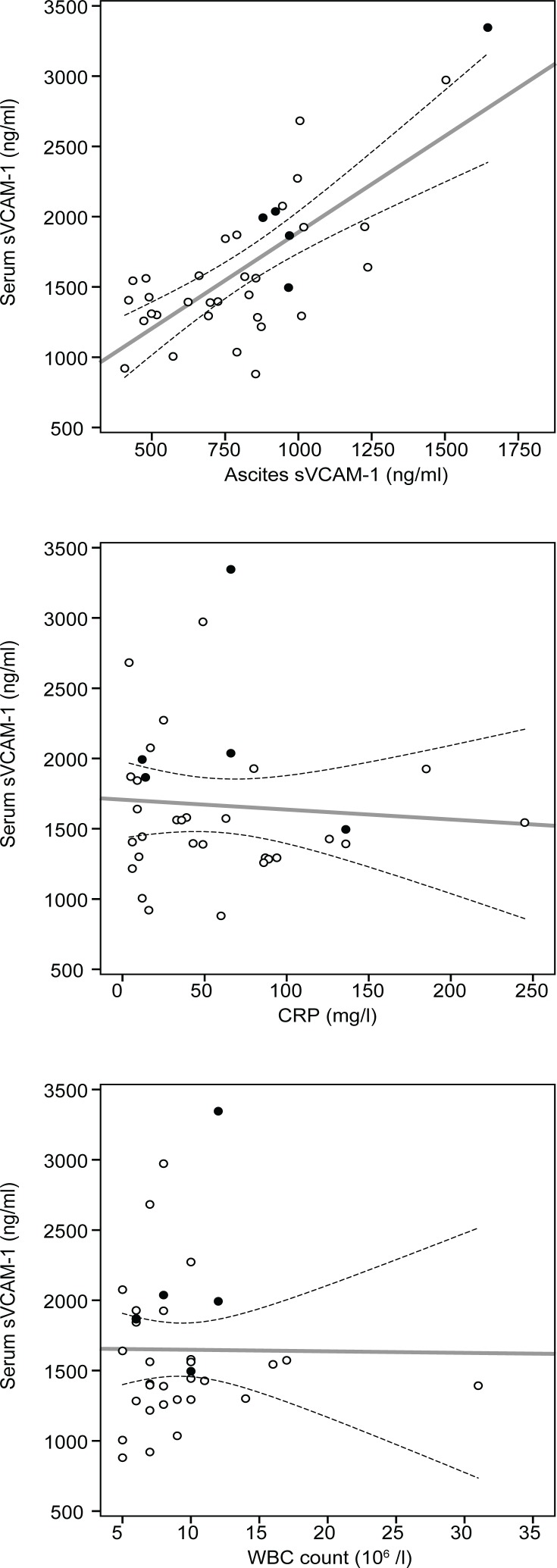
Correlation between serum sVCAM-1 concentrations and sVCAM-1 concentrations in ascites, C-reactive protein (CRP) level and white blood cell (WBC) count. Dashed lines represent 95% confidence intervals for the regression line. Black dots represent patients who died from ovarian cancer.

The mean concentration of sVCAM-1 in ascites was significantly lower (two fold) than that in serum (Table 1). A significant positive correlation between sVCAM-1 concentrations in serum and ascites was observed (*r* = 0.733, *p* < 0.001) (Figure 1).

Thirty-four (90%) patients had elevated CRP levels. The mean CRP value was 59.8 ± 56.6 mg/l. Thirteen (36%) patients had elevated WBC levels. The mean WBC count was 13.3 ± 5.8 x 10^6^ /L. There was no correlation between serum sVCAM-1 concentration and each of CRP and WBC levels (Figure 1).

When sVCAM-1 concentrations were compared with standard clinicopathologic variables, only serum sVCAM-1 concentrations were weakly correlated to tumour size by the Spearman test (*r* = 0.347; *p* = 0.038). Concentrations of sVCAM-1 in neither ascites nor serum were correlated with FIGO stage or tumour grade (data not shown).

During the mean follow-up period of 11 months (range 0–23) from the time of surgery, 5 (14%) patients died from ovarian cancer, 4 out of 5 before chemotherapy was started. We therefore evaluated the association of VCAM-1 concentrations in serum and ascites with patient outcome. In view of the small number of patients included and the short period of follow-up, only univariate analysis was performed. Our intention was to obtain preliminary data on whether sVCAM-1 has potential as a prognostic factor and, if so, to evaluate it in the future. Higher concentrations of sVCAM-1 in serum and ascites turned out to be associated with poor outcome, since there was a significant difference in sVCAM-1 serum concentration between the 31 patients who are still living and the 5 who did not survive; 1557.4 ± 470 ng/ml versus 2147.2 ± 702.8 ng/ml (*p* = 0.02) (Table 2). The same is also true for sVCAM-1 concentrations in ascites: 776.2 ± 264.4 ng/ml for those who are still living versus 1076.5 ± 320.4 ng/ml for those who died (*p* = 0.028). Contrary to our expectation, none of the other variables included in the univariate analysis (age, tumour size, tumour grade, FIGO stage) were correlated with patient outcome (Table 2).

## Discussion

Blood assay for detecting tumour biomarkers is an important non-invasive method for establishing cancer diagnosis, patient prognosis and treatment outcome.^20^ One promising candidate for an OC biomarker, as demonstrated by a recent study, is the sialoglycoprotein VCAM-1.^17^–^21^ Its soluble form sVCAM-1 has been detected in serum but its origin is not well investigated. We therefore performed a study that might elucidate the relationship of sVCAM-1 in two different environments: ascites as the local environment, to which VCAM-1 is shed, and serum, where other influences that determine the steady-state levels of sVCAM-1, could be expected (*e.g.,* systemic inflammation). The concentration of sVCAM-1 in ascites of OC patients has not been measured yet. Additionally, we examined the relationship of sVCAM-1 concentrations to various clinicopathologic variables, as well as to patient outcome. The major new findings in our study are as follows: (1) the sVCAM-1 concentration in ascites is approximately half of that in serum; (2) ascites is an important source of sVCAM-1 in systemic circulation, since serum sVCAM-1 concentrations are positively correlated to ascites sVCAM-1 concentrations; (3) serum sVCAM-1 concentrations are not correlated to CRP and WBC levels, so elevated serum sVCAM-1 levels are probably not the result of systemic inflammation; (4) serum sVCAM-1 but not ascites concentrations are weakly correlated to tumour size, which might suggest that sVCAM-1 originating from the primary tumour can at least partly reach serum directly and not through ascites; (5) higher concentrations of sVCAM-1 are associated with patient death from OC in a short period after surgery, in almost all cases (4 out of 5) before chemotherapy was started. This might indicate a relation of sVCAM-1 to an aggressive form of the disease.

The detected sVCAM-1 concentration in ascites reflects its production in the local tumour environment. The cellular fraction of ascites consists mainly of OC cells, lymphocytes and mesothelial cells.^12^ The expression of a membrane-bound form of VCAM-1 in OC patients’ peritoneal biopsies and ascites has been shown in activated mesothelial cells, monocytes/macrophages, and rarely in T cells.^15^–^22^ We therefore assume that the main cellular sources of sVCAM-1 in ascites are probably mesothelial cells and monocytes/macrophages. VCAM-1 expressed in activated mesothelial cells has been identified as an important mediator of adhesion of OC cells to and invasion through the mesothelium.^15^ An increased concentration of sVCAM-1 in ascites could therefore indicate a biologic potential of disease progression to highly invasive tumours, growing under the mesothelium, which is associated with a poor prognosis.^15^ Whereas the mechanism that regulates VCAM-1 expression in mesothelial cells in OC patients is unknown, a known inducer of VCAM-1 expression, tumour necrosis factor α (TNF-α), is expressed by OC cells, macrophages, and is found in ascites.^23^,^24^ Increased production of sVCAM-1 in ascites could also be due to enhanced proteolytic cleavage from the cell surface. VCAM-1 shedding from the cell surface *in vitro* can be mediated by two distinct metalloprotease activities: a constitutive VCAM-1 sheddase that is active under normal conditions, and inducible protease identified as a tumour necrosis factor-α-converting enzyme (TACE or ADAM 17).^25^ The significance of VCAM-1 shedding *in vivo* is not yet known, but at least two roles are possible. First, cleavage of VCAM-1 may play a role in regulation of its adhesive function by rapidly decreasing its levels at the cell surface. A second potential role is that cleavage near the transmembrane region leads to the release of intact sVCAM-1, which may remain functionally active.^25^

Using Pearson correlation test, we were able to confirm a positive correlation between ascites and serum sVCAM-1 concentrations. This result reflects the important contribution of sVCAM-1 from ascites to increasing the concentration of sVCAM-1 in serum, which is possibly because of the direct access of ascites from the peritoneum to circulation. Ascites carrying soluble proteins can enter the subperitoneal lymphatic lacunae and the lymphatic fluid finally drains into the left subclavian vein.^26^ The concentration of sVCAM-1 in ascites is approximately half of the concentration in serum, assuming that all of the sVCAM-1 from ascites reached the blood. Other sources of sVCAM-1 in serum should therefore be considered. The existence of high basal levels of sVCAM-1 in serum of healthy people suggests a likely physiological role.^27^ Another source might be sy*s*temic inflammation, as an inherent component in cancer patients.^19^ Inflammatory cytokines markedly induce VCAM-1 expression in endothelial cells. Endothelial VCAM-1 functions by regulating leukocyte attachment and extravasation at sites of inflammation, a process that is similar to invasion of OC cells through the mesothelium.^28^ Inflammatory markers (CRP and WBC) were elevated in our population, providing confirmatory evidence of a connection between the inflammation process and carcinogenesis.^19^,^29^,^30^ The results of our study showed no correlation between sVCAM-1 and inflammatory markers, so an influence of systemic inflammation on increased sVCAM-1 can be excluded.

Among standard clinicopathologic characteristics, only tumour size was weakly correlated with sVCAM-1 in serum. Tumour size was not in correlation with sVCAM-1 concentration in ascites, indicating that sVCAM-1 originating from the primary tumour, could at least partly reach serum directly. sVCAM-1 could exit from the primary tumour through new micro vessels functionally connected to the peripheral blood circulation. Without angiogenesis, tumour expansion cannot proceed beyond 1–2 mm, and invading blood vessels occupy 1.5% of the tumour volume.^31^ We speculate that the origin of sVCAM-1 might be perivascular cells in developing vessels. VCAM-1 expressed in perivascular cells and integrin α4β1 in endothelial cells mediate adhesion between these two types of cells, an event that is required for the survival of proliferating endothelial and perivascular cells and, therefore, for neovascularization. VCAM-1 is expressed only by proliferating and not by quiescent perivascular cells.^32^ The growth of peritoneal metastasis has also been reported to be dependent on neovasculature.^31^,^33^ An explanation of the weak correlation between tumour size and serum sVCAM-1 concentration in our study might be that only the size of the primary tumour was included in the comparison and not the extent of peritoneal metastasis. A direct measurement of the degree of neoangiogenesis by a technique such as microvessel density would yield a more conclusive result.

Serum and ascites sVCAM-1 concentrations were not in correlation with other clinicopathologic variables, such as FIGO stage (III versus IV), grade of differentiation and histological tumour type. Huang *et al.* also found no correlation between VCAM-1 and FIGO stage in OC patients in their study but they examined the expression of VCAM-1 in ovarian cancer tissue samples and not in human fluids.^21^ A possible explanation could be that sVCAM-1 in the two fluids does not reflect the stabilized spread of the disease defined by stage but by the biologic potential of ovarian cancer to metastasis. In support of this hypothesis, we found that patients with more aggressive disease, who did not survive, had significantly higher levels of sVCAM-1 in both serum and ascites, although we are aware that the number of cases in our sample is not sufficient to confirm the predictive potential of sVCAM-1 for different biologic behaviour. More extensive research should be done to prove this assumption. However, one recently published study showed that VCAM-1 overexpression in OC cells was an independent predictor of overall survival in 251 OC patients, thereby providing support for our result.^21^ VCAM-1 in this study was localized in the cytoplasm of tumour and stromal cells and not on the cell surface. How the intracellular form of VCAM-1 is related to sVCAM-1 in body fluids is as yet unknown. A limitation of our study was the small number of patients included, which prevented reliable analysis of subgroups, especially in the case of histological tumour type.

In conclusion, the present study demonstrates that serum concentrations of sVCAM-1 in advanced stage OC patients correlate with sVCAM-1 concentrations in ascites, that represents tumour local environment, and not with systemic inflammation, thus expressing the biologic potential of malignant disease to metastasis. Another important practical implication is that higher serum and ascites sVCAM-1 concentrations are correlated with worse patient outcome, which supports the predictive potential of sVCAM-1 for different biologic behaviour of OC, which needs to be proven in more extensive research.

## Figures and Tables

**TABLE 1.** Clinical characteristics of the patients and laboratory data

**Variables**	**Data**
No. of patients	36
Age, years	
Mean	59.9 ± 12.1
Range	28–83
**Vital status for the follow up period of 11,2 monthsn (%)**	
Dead	5 (13.9 %)
Alive	31 (86.1 %)
FIGOstage, *n* (%)	
III	26 (72.2 %)
IV	10 (27 %)
**Grade of differentiation, n (%)**	
3	19 (52.8 %)
2	11 (30.5 %)
1	5 (13.9 %)
NA	1 (2.8 %)
**Hystological type**	
serous carcinoma	29 (80.5 %)
endometroid carcinoma	3 (8.3 %)
mixed type and clear cell	4 (11.1 %)
Tumour size, cm	14.2 ± 6.8
WBC, ×10^6^ /l	9.28 ± 4.7
CRP, mg/l	55.1 ± 56.2
Serum sVCAM-1, ng/ml	1639.3 ± 537.2
Ascites sVCAM-1, ng/ml	817.9 ± 287.7
Ratio ascites/serum, range	0.51 ± 0.15 (0.30 – 0.97)

FIGO = International Federation of Gynecology and Obstetrics; NA = not avalible; CRP = C-reactive protein; WBC = white blood cells; sVCAM-1 = soluble Vascular Cell Adhesion Molecule-1

**TABLE 2.** Univariate analysis of OC prognostic factors for cancer-specific survival

**Variables**	**Dead (n=5)**	**Alive (n=31)**	p^*^
**Continuous**			
Age, years	69.0 ± 8.6	58.5 ± 12.1	0.072
Serum sVCAM-1, ng/ml	2147.2 ± 702.8	1557.5 ± 470.1	0.02
Ascites sVCAM-1, ng/ml	1076.5 ± 320.4	776.2 ± 264.4	0.028
Tumour size, cm	18.0 ± 9.3	13.3 ± 6.1	0.145
**Categorical Tumour grade, n**	(n=4)	(n=31)	0.588
1	0	5	
2	1	10	
3	3	16	
**FIGO stage, n**	**(n=5)**	**(n=31)**	**0.511**
III	3	23	
IV	2	8	

FIGO = International Federation of Gynecology and Obstetrics; sVCAM-1 = soluble Vascular Cell Adhesion Molecule-1;

* Independent t test for continuous variables and chi-square test for categorical ones.
